# Assessment of the Operational Aspects of Health and Wellness Centers in Randomly Selected Blocks of Eastern India

**DOI:** 10.7759/cureus.76445

**Published:** 2024-12-26

**Authors:** Nidhi Prasad, Sanjay Kumar, Dharmvir Ranjan Bharati

**Affiliations:** 1 Community Medicine, Indira Gandhi Institute of Medical Sciences, Patna, IND

**Keywords:** ayushman bharat, comprehensive primary healthcare, from india, health and wellness center, health promotion

## Abstract

Introduction

Launched in 2018, by the Government of India, the Health and Wellness Centers (HWCs) initiative under the Ayushman Bharat program aims to transform India's primary healthcare system. This study evaluates the functioning of Health and Wellness Centres (HWCs) in random blocks of Patna, Bihar, focusing on service availability, infrastructure, manpower challenges, and overall effectiveness in meeting healthcare needs.

Methodology

The assessment aimed to understand the current state of functioning of 16 HWCs and identify areas for improvement through a Strengths, Weaknesses, Opportunities, Threats (SWOT) analysis. This evaluation was conducted via face-to-face interviews with the senior-most personnel at each facility and assessments of various components within the centers.

Results

The results indicate that while routine immunization services are commendably available at 81.25% and outpatient services at 68.75%, significant gaps persist, particularly in child and adolescent care, emergency services, mental health management, and non-communicable disease (NCD) screening, which is limited to only 18.75% of centers. Infrastructure issues exacerbate these challenges, with 75% of centers operating in inadequate buildings lacking essential amenities. Staffing shortages affect 68.75% of facilities, leading to compromised service continuity, especially in urban areas. Alarmingly, pediatric and adolescent services are available in only 6.25% of centers, highlighting a critical need for targeted healthcare for younger populations.

Conclusion

Despite these challenges, the assessment underscores the necessity for strategic interventions in public health policy to address identified gaps, enhance service delivery capabilities, and improve community engagement through wellness initiatives. Expanding teleconsultation services can also bridge access gaps for underserved populations. Overall, addressing these issues is vital for strengthening the healthcare system in Patna and ensuring comprehensive health coverage for all residents.

## Introduction

The Health and Wellness Centers (HWCs) initiative under the Ayushman Bharat program aims to transform India's primary healthcare system. Launched in 2018 by the Government of India, this ambitious program seeks to provide comprehensive healthcare services to address both preventive and curative needs. HWCs serve as the cornerstone of India’s health system, focusing on wellness and disease prevention while also providing essential medical care [1]. 

Recently, the Government of India introduced significant changes to HWCs under the Ayushman Bharat initiative, aiming to enhance healthcare delivery and accessibility. As part of a strategic initiative, these health centers have been rebranded as "Ayushman Arogya Mandir," embracing a holistic approach to healthcare. The tagline "Arogyam Parmam Dhanam" - a profound Sanskrit phrase - beautifully encapsulates the significance of health, translating to "health is the ultimate wealth" [2].

This rebranding effort underscores the importance of prioritizing health and wellness while fostering a culture of comprehensive care. However, there is a notable disparity in the establishment of HWCs, with high-income states like Tamil Nadu, Gujarat, Andhra Pradesh, and Maharashtra having the highest number of operational centers [3]. The initiative holds particular significance in Patna, where the public healthcare system has seen considerable evolution over the past few decades. Despite these ambitious goals, the implementation of HWCs faces challenges specific to local contexts. For instance, in Patna, the overlap between various health institutions creates coordination difficulties that can hinder effective rollout. This indicates an uneven distribution of resources and healthcare infrastructure across different regions.

The study aimed to assess the infrastructure readiness and availability of human resources, healthcare services, and essential drugs at Health and Wellness Centres (HWCs) in the western block of Patna district. Specifically, the study aimed to evaluate the availability of manpower, an expanded range of comprehensive primary healthcare services, diagnostics, medicines, teleconsultation facilities, and wellness components. By analyzing these parameters, the study seeks to provide valuable insights for program managers and primary care practitioners to strengthen the implementation of Comprehensive Primary Healthcare (CPHC) initiatives.

## Materials and methods

A cross-sectional study was conducted from January 2021 to December 2022 across HWCs in Patna. Data were collected using a predesigned questionnaire based on Ayushman Bharat guidelines. Ethical clearance was obtained from the Institutional Ethics Committee of IGIMS, Patna (reference no. #320/IEC/IGIMS/2021, dated December 13, 2021). The evaluation involved a systematic assessment of 16 HWCs in the western block of Patna. A list of the primary health centers (PHC) and sub-centers (SC) that were upgraded to HWC was obtained in 24 blocks and through random sampling these 16 HWC were chosen. These were the centers that were upgraded from health sub-centers and primary health centers to HWC. The principal investigator visited Health and Wellness Centers (HWCs) under the PHC. Data were gathered through site visits, interviews were conducted with healthcare providers like community health officers (CHOs), auxiliary nurse midwives (ANMs), multi-purpose workers (MPWs), and medical officers based on checklists, and a comprehensive review of the secondary data was conducted for both types of facilities (Table 1).

**Table 1:** Criteria for assessment. IEC: Information, Education, and Communication

1	Infrastructure and resources	Examination of the physical condition of buildings, availability of essential amenities, and diagnostic equipment
2	Manpower	Evaluation of the availability and qualifications of medical officers, nurses, and other healthcare staff
3	Service availability	Analysis of the range of services offered, including routine immunization, outpatient care, antenatal care, family planning, and specialized services
4	Diagnostic capabilities	Availability and functionality of basic and advanced diagnostic tests
5	Wellness and IEC activities	Implementation of wellness programs and availability of IEC materials
6	Teleconsultation services	Availability and utilization of teleconsultation facilities

The data were collected using a predesigned questionnaire according to the proforma for Ayushman-Bharat Health and Wellness Centre facility survey guidelines. The collected data were analyzed to determine the strengths and weaknesses of the HWCs in terms of service delivery and overall healthcare provision. The assessment was also done to check whether the health centers were operational or not. The data collected from various variables across each domain were meticulously recorded and entered on a Microsoft Excel spreadsheet for further processing and analysis. This systematic approach enabled the research team to organize, visualize, and examine the data in a comprehensive and efficient manner. The data were also analyzed critically to determine the strengths and weaknesses of the HWCs in terms of service delivery and overall healthcare provision.

## Results

The analysis shows that care in pregnancy and childhood is provided by nine out of 16 centers, representing 56.25% (n=9). This is a positive indicator of maternal and child health services in the area. However, neonatal and infant healthcare services are alarmingly low, with only two centers (12.50%; n=2) offering these essential services. Similarly, childhood and adolescent healthcare services are also limited to just two centers (12.50%; n=2). Family planning services are available at eight centers (50.00%; n=8), which reflects a moderate level of support for reproductive health, while general outpatient care is offered at nine centers (56.25%; n=9). Management of communicable diseases is provided at only one center (6.25%; n=1), while emergency medical services and mental health management are absent altogether, with 0% availability reported for both services. Additionally, screening for non-communicable diseases (NCDs) is limited to six centers (37.50%; n=6), highlighting a lack of proactive measures to address the rising burden of these diseases as shown in Table 2.

**Table 2:** Service availability across the various health and wellness center. The data have been presented as percentage.

Service type	Number of centers providing	Total centers	Percentage
Care in pregnancy and childhood	9	16	56.25
Neonatal and infant healthcare services	2	16	12.50
Childhood and adolescent healthcare services	2	16	12.50
Family planning services	8	16	50.00
Management of communicable diseases	1	16	6.25
General outpatient care	9	16	56.25
Screening for non-communicable diseases	6	16	37.50
Basic oral healthcare	1	16	6.25
Emergency medical services	0	16	0.00
Mental health management	0	16	0.00

The assessment also identified several challenges affecting service delivery across the HWCs. Infrastructure and resource issues were reported at 12 out of 16 centers (75.00%; n=12), indicating that many facilities operate in inadequate conditions lacking essential amenities. Manpower challenges affect 11 centers (68.75%; n=11), which significantly impacts continuity in service delivery.

The limited implementation of wellness programs at only three centers (18.75%; n=3) and Information, Education, and Communication (IEC) activities at 43.75% (n=7) centers reflect a critical area for improvement in promoting preventive health measures within communities. Teleconsultation services are available at just one center (6.25%; n=1), indicating underutilization of technology that could enhance healthcare access in underserved areas as shown in Table 3.

**Table 3:** Challenges faced by centers affecting service delivery. The data have been presented as percentage.

Aspect	Number of centers affected	Total centers	Percentage
Infrastructure and resources	12	16	75.00
Manpower challenges	11	16	68.75
Service availability	13	16	81.25
Diagnostic capabilities	11	16	68.75
Wellness activities	14	16	87.5

The study highlights both strengths and weaknesses in healthcare delivery. A significant strength is the high availability of routine immunization services at 81.25% (n=13), which is crucial for preventing vaccine-preventable diseases. Additionally, outpatient services are accessible at 68.75% (n=11) of centers, providing essential primary care. However, critical weaknesses exist, particularly in pediatric services, which are available at only one center (6.25%; n=1), and the complete absence of emergency medical services.

Teleconsultation services are provided by only one center (6.25%; n=1), indicating a significant area for improvement in healthcare access. Population enumeration is conducted at three centers (18.75%; n=3), while only five centers (31.25%; n=5) have filled out Community-Based Assessment Checklist (CBAC) forms. Notably, cancer screening is absent across all centers (0.00%), highlighting a major gap in preventive health services. Information, Education, and Communication (IEC) materials are available at seven centers (43.75%; n=7), and family planning services are offered at four centers (25%; n=4). Non-communicable disease (NCD) screening is limited to three centers (18.75%; n=3). Rapid tests for pregnancy, hemoglobin, and blood sugar are available at 11 centers (68.75%; n=11), but advanced diagnostic tests and functional complete blood count (CBC) machines are present in only one center each (6.25%; n=1) as mentioned in Table 4.

**Table 4:** Centers providing the service delivery. *Community based assessment checklist. The data have been presented as percentage.

Components of services	Number of centers providing services	Total centers	Percentage
Teleconsultation	1	16	6.25
Population enumeration	3	16	18.75
CBAC form filled*	5	16	31.25
Cancer screening	0	16	0.00
IEC material	7	16	43.75
Family planning services	4	16	25
Non-communicable disease (NCD) screening	3	16	18.75
Rapid tests (pregnancy, hemoglobin, blood sugar)	11	16	68.75
Advanced diagnostic tests	1	16	6.25
CBC machine functional	2	16	12.50

**Figure 1:**
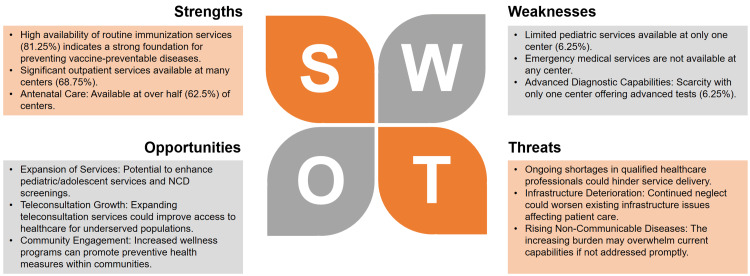
Strengths, Weaknesses, Opportunities, Threats (SWOT) analysis. The image is generated by the authors of this study.

Opportunities for improvement include enhancing pediatric and adolescent healthcare services and expanding screenings for non-communicable diseases (NCDs). Yet, these opportunities are threatened by ongoing shortages of qualified healthcare professionals, which could hinder effective service delivery. Infrastructure issues also pose a challenge, with 75% (n=12) of centers operating in inadequate conditions lacking essential amenities as shown in Figure 1.

## Discussion

The assessment of Health and Wellness Centers (HWCs) in the western block of Patna reveals a complex landscape of healthcare delivery characterized by both notable strengths and significant challenges. This discussion elaborates on the findings, emphasizing the implications for public health policy and the need for strategic interventions to enhance service delivery. While there is a robust foundation for primary healthcare through immunization services, critical gaps exist in child and adolescent care, emergency services, mental health management, NCD screening capabilities including cancer, and advanced diagnostics [4].

Infrastructure issues further complicate service delivery; a significant number of centers (75%) operate in inadequate buildings lacking essential amenities. Five of the centers were housed in independent buildings, while the rest were either collocated or operating in private buildings, as in Punjab [5]. Many facilities are housed in donated or personal buildings, with some nearly non-functional due to poor maintenance. Branding was present in almost two-thirds of the centers, as reported in a study that also found 90% of the centers had branding done [6]. Addressing manpower challenges is essential for continuity in service delivery; strategic recruitment efforts must be prioritized alongside infrastructure improvements to ensure safe environments for patients [7,8].

One of the notable strengths identified in the assessment is the high availability of routine immunization services, reported at 81.25%, alongside outpatient services at 68.75%. These figures indicate a solid foundation for primary healthcare, essential for preventing common diseases and providing immediate care for minor ailments, which is crucial for community health. The effective outreach efforts and community engagement reflected in these statistics are vital for building trust in public health systems. Furthermore, antenatal care (ANC) services are accessible at 62.5% of centers, ensuring that many pregnant women receive necessary prenatal care. Family planning services are offered by 50% of the centers, reflecting a moderate level of support for reproductive health [9].

Manpower challenges are another critical issue affecting service delivery continuity. Staffing shortages impact 68.75% of centers, with many healthcare workers often assigned additional duties elsewhere, leading to burnout and reduced quality of care. The issue was very grave in urban centers as compared to rural centers as compared to the study done in Odisha [9]. The COVID-19 pandemic exacerbated these staffing challenges as personnel were deployed away from their primary postings without adequate replacements [10]. This situation has resulted in compromised service delivery and highlights the urgent need for strategic recruitment and retention efforts alongside investments in training programs.

One major concern is the alarmingly low availability of pediatric and adolescent services, which stands at only 6.25%. This gap indicates a lack of targeted healthcare for younger populations, which is critical given the rising health issues among children and adolescents. Additionally, screening for non-communicable diseases (NCDs) is limited to just 18.75% of centers, raising concerns about the capacity to address the growing burden of these diseases in India. Expanding these services is essential for comprehensive healthcare.

The assessment also highlights deficiencies in advanced diagnostic capabilities, with such tests available in only 6.25% of centers. Basic diagnostic services also show promise, with rapid tests for pregnancy, hemoglobin, and blood sugar available at 68.75% of centers which was also done in Chhattisgarh [6]. This availability contributes to early detection and management of health conditions, which is vital for improving overall health outcomes. However, despite these strengths, the assessment reveals significant gaps in service delivery that warrant immediate attention as the machines were non-functional due to the deputation of staff at other places who had not returned to their place of appointment and no one was there to replace them. This limitation restricts the ability to accurately diagnose complex health conditions, underscoring the need for better-equipped facilities capable of providing comprehensive diagnostic services.

The limited implementation of wellness programs (18.75%) and Information, Education, and Communication (IEC) activities (43.75%) reflects a critical area for improvement in promoting preventive health measures within communities. Regular wellness initiatives are essential for educating the population about healthy practices and encouraging proactive health-seeking behaviors. E-Sanjeevani OPD for online consultation was done at one of our centers which was also mentioned as the best practice in Bhandara district [11].

Teleconsultation services are available at only 6.25% of centers, indicating the underutilization of technology that could enhance healthcare access, especially in underserved areas where mobility may be a barrier to receiving care. Expanding teleconsultation capabilities can bridge gaps in healthcare access and improve service delivery efficiency [12,13].

The strengths of this study lie in its detailed assessment of Health and Wellness Centers (HWCs), providing a comprehensive understanding of their capabilities and challenges. It highlights the robust foundation of primary healthcare services, such as routine immunization, antenatal care, and outpatient services, which are essential for preventive and curative care. The study also recognizes the implementation of innovative solutions like teleconsultation and identifies effective community engagement efforts. By showcasing best practices and strengths in service delivery, the findings offer valuable insights for enhancing healthcare infrastructure, addressing manpower challenges, and optimizing resource allocation to improve overall public health outcomes.

The implications for public health policy stemming from these findings are significant. There is a pressing need to allocate resources strategically to address identified gaps in pediatric care, NCD screening, advanced diagnostics, and infrastructure improvements. Addressing manpower challenges through targeted recruitment and retention strategies will be essential to enhance service delivery capabilities.

Moreover, engaging communities through wellness programs and IEC activities can empower individuals to take charge of their health while fostering trust in public health systems. Expanding teleconsultation services can further bridge gaps in healthcare access for populations residing in remote areas.

However, the study is based on a relatively small sample size of only 16 centers, which may not be representative of the broader healthcare landscape in Bihar or India as a whole. This limited scope can lead to skewed results that do not capture the full range of challenges and strengths across different regions. While the article identifies significant gaps in services such as emergency medical care and mental health management, it does not provide a detailed analysis of the underlying factors contributing to these deficiencies. Understanding the root causes, such as policy limitations, funding constraints, or socio-cultural barriers, would be essential for developing effective interventions to improve healthcare delivery.

## Conclusions

The assessment reveals that while HWCs in the western block of Patna provide essential primary healthcare services, there are notable gaps that need addressing to enhance healthcare quality and outcomes in the region. By focusing on strengthening infrastructure, enhancing service availability, addressing manpower shortages, and promoting community engagement through wellness initiatives, policymakers can ensure that HWCs fulfill their potential as effective providers of comprehensive healthcare under the Ayushman Bharat initiative. Addressing these identified gaps effectively requires strategic investments in infrastructure improvements, recruitment of qualified healthcare professionals, expansion of emergency medical services, and increased access to diagnostic capabilities to ensure that all community members receive the care they need. Community engagement is crucial for improving perceptions about these facilities based on past experiences with health services. These findings underscore the need for strategic enhancements in service delivery, particularly in telehealth, cancer screening, and comprehensive diagnostic capabilities to improve overall healthcare outcomes in the region.
